# Supplementary material for: Genome-Scale Metabolic Modeling with Protein Expressions of Normal and Cancerous Colorectal Tissues for Oncogene Inference
Source: Metabolites. 2019 Dec 25;10(1):16. doi: 10.3390/metabo10010016 (PMC7022839; doi:10.3390/metabo10010016)
Supplement: Supplementary file 1 [file metabolites-10-00016-s001.zip › SupplementaryFile1.pdf]

Supplementary Materials for

Genome-Scale Metabolic Modeling with Protein Expressions of Normal and Cancerous Colorectal Tissues for Oncogene Inference

Feng-Sheng Wang*, Wu-Hsiung Wu, Wei-Shiang Hsiu, Yan-Jun Liu and Kuan-Wei Chuang

Department of Chemical Engineering, National Chung Cheng University, Chiayi, Taiwan

*Corresponding author

chmfsw@ccu.edu.tw

Table S1: Nutrients from RPMI-1640 and DMEM medium. Please refer to the Nutrients worksheet in the SupplementaryFile2.xlsx file.

Table S2: Genes associated to FAP and HNPCC from GeneCards database. Please refer to the EnzymeGenes worksheet in the SupplementaryFile2.xlsx file.

Table S3: Average change ratios (CR) and similarity ratios (SR) of oncogenes in different GSMNs.

	M1		M2		M3		M4		M5	
**Gene**	**Ave. CR**	**Ave. SR**	**Ave. CR**	**Ave. SR**	**Ave. CR**	**Ave. SR**	**Ave. CR**	**Ave. SR**	**Ave. CR**	**Ave. SR**
CAT	0.934	0.982	0.87	0.955	0.897	0.966	0.871	0.958	0.847	0.938
GPI	0.931	0.981	0.867	0.956	0.886	0.969	0.835	0.955	0.835	0.937
PPA2	0.935	0.982	0.869	0.954	0.849	0.961	0.865	0.958	0.847	0.938
HMGCL	0.935	0.982	0.869	0.955	0.851	0.961	0.865	0.958	0.317	0.809
AGXT	0.933	0.982	0.869	0.954	X	X	0.867	0.957	0.837	0.938
GLRX2	0.932	0.982	0.869	0.954	0.897	0.965	0.861	0.955	0.837	0.938
GRHPR	0.934	0.982	0.869	0.954	0.562	0.877	0.865	0.955	X	X
G6PD	0.827	0.98	0.868	0.954	0.87	0.962	0.83	0.955	0.834	0.938
H6PD	0.918	0.982	0.866	0.954	0.891	0.961	0.865	0.957	0.835	0.938
G6PC3	0.936	0.982	0.868	0.954	0.588	0.876	0.425	0.846	0.214	0.802
SLC26A6	0.934	0.982	0.869	0.952	0.852	0.96	0.865	0.958	0.834	0.939
SLC37A4	0.93	0.982	0.868	0.954	0.883	0.959	0.864	0.957	0.836	0.938
SLC9A1	0.932	0.982	0.863	0.953	0.898	0.971	0.864	0.958	0.834	0.938
MLYCD	0.933	0.982	0.868	0.954	0.42	0.866	0.446	0.859	0.171	0.72
PYCR3	0.934	0.982	0.644	0.888	NA	NA	0.773	0.922	0.638	0.907
PRODH2	0.933	0.981	0.868	0.954	X	X	0.758	0.933	0.839	0.937
IMPDH1	0.934	0.982	0.865	0.953	0.71	0.915	0.668	0.904	0.322	0.841
CYBRD1	0.934	0.981	0.869	0.954	0.85	0.961	0.861	0.955	0.837	0.938
CDO1	0.934	0.982	0.869	0.954	0.897	0.965	0.704	0.91	0.837	0.938
LIPC	0.940	0.981	0.869	0.954	0.853	0.961	0.871	0.958	NA	NA

M1: GSMN reconstructed by the CORDA algorithm taking the Recon 2.2 general model and HPA protein expression data as input.
M2: GSMN reconstructed by the CORDA algorithm taking the Recon 2.2 general model and TCGA gene expression data as input.
M3: GSMN reconstructed by the CORDA algorithm taking the Recon 2.02 general model and HPA protein expression data as input.
M4: GSMN reconstructed by the CORDA algorithm taking the Recon 3D general model and HPA protein expression data as input.
M5: GSMN reconstructed by the iMAT algorithm taking the Recon 2.2 general model and HPA protein expression data as input.
X means the gene is not included in the reconstructed model.

Figure S1: A numerical example to illustrate the computation of template, similarity ratio, and LFC.

- Suppose that a toy metabolic network consisted of 5 metabolites, and their synthesis rates for the normal model, cancer model and three mutant models are shown as follows:

Synthesis rate for each metabolite:

$$r_m = \sum_{k \in \Omega^c} \left(\sum_{N_{ij} > 0, j} N_{ij} v_{f,j} - \sum_{N_{ij} < 0, j} N_{ij} v_{b,j} \right), m \in \Omega^m$$

Flux Pattern					
	Normal	Cancer	Mutant 1	Mutant 2	Mutant 3
Metabolite 1	1	4	3.4822	3.249	3.321
Metabolite 2	0.5	1.4142	0.3299	1.1487	1.3193
Metabolite 3	1	0.7071	0.7579	0.8123	0.933
Metabolite 4	0.5	0.25	0.2872	1.1487	0.4353
Metabolite 5	1	1.4142	1.36	0.7579	1.0353

- The logarithmic fold changes (LFC_m) for the template and each mutant are computed as follows:

$LFC_m^T = \log_2\left(r_{m,\text{cancer}} / r_{m,\text{normal}}\right)$		$LFC_m = \log_2\left(r_{m,\text{mutant}} / r_{m,\text{normal}}\right)$		
	Template	Mutant 1	Mutant 2	Mutant 3
Metabolite 1	2	1.8	1.7	1.9
Metabolite 2	1.5	-0.6	1.2	1.4
Metabolite 3	-0.5	-0.4	-0.3	-0.1
Metabolite 4	-1	-0.8	1.2	-0.2
Metabolite 5	0.5	0.45	-0.4	0.05

- Suppose that we set 3% of the tolerance for increase/decrease, i.e. $tol_+ = 0.0426$ and $tol_- = -0.0439$
- The similarity indicator, $\mu_m^{T/Trxn}$, and similarity ratio, $SR^{T/Trxn}$ are as follows:

Similarity indicator:

$$\mu_m^{T/T_{rxn}} = \begin{cases} 1, \text{ if } LFC_m > tol_+ \text{ and } LFC_m^{T/T_{rxn}} > tol_+ \\ -1, \text{ if } LFC_m < tol_- \text{ and } LFC_m^{T/T_{rxn}} < tol_- \\ 0, \text{ otherwise} \end{cases}$$

	Similarity indicator		
	Mutant 1	Mutant 2	Mutant 3
Metabolite 1	1	1	1
Metabolite 2	0	1	1
Metabolite 3	-1	-1	-1
Metabolite 4	-1	0	-1
Metabolite 5	1	0	1
$SR^{T/T_{rxn}} = \sum_{m=1}^{5} \left| \mu_m^{T/T_{rxn}} \right| \Big/ 5$	0.8	0.6	1.0

- The logarithmic fold change is evaluated through the membership grades defined as follows:

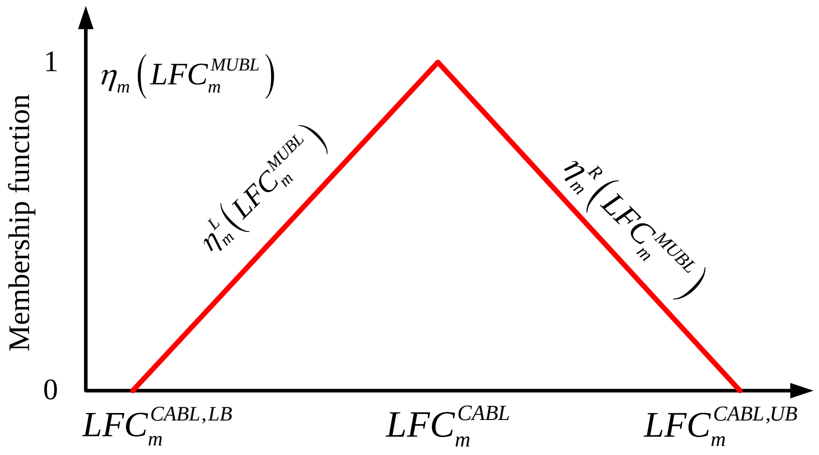

	Mutant 1	Mutant 2	Mutant 3
Metabolite 1	0.9	0.85	0.95
Metabolite 2	0	0.8	0.93
Metabolite 3	0.8	0.6	0.2
Metabolite 4	0.8	0	0.2
Metabolite 5	0.9	0	0.1
Mean	0.68	0.45	0.476

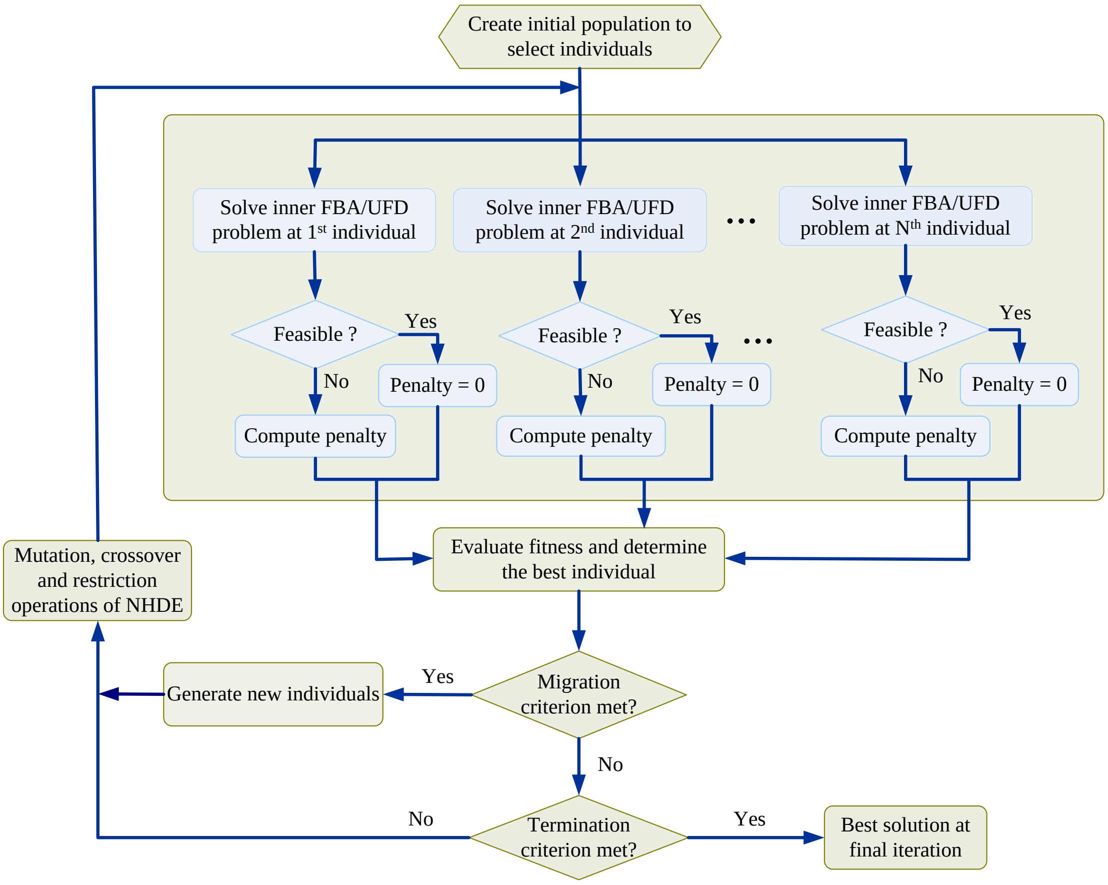

Figure S2: Flowchart of the NHDE algorithm for solving TLOP. The NHDE algorithm is applied to determine the outer decision variables and objective function. The inner optimization problems, HBA and UFD, are a parallel computation. Each FBA problem is solved through a linear programming solver to obtain the maximum cellular objective value. The maximum objective provides as a constraint for the UFD problem solved by a quadratic programming to obtain the uniform flu distribution for each mutant. The mutant flux pattern is then applied to compare with the template in order to evaluate the fitness in NHDE for iteratively evolving to yield the best mutant.

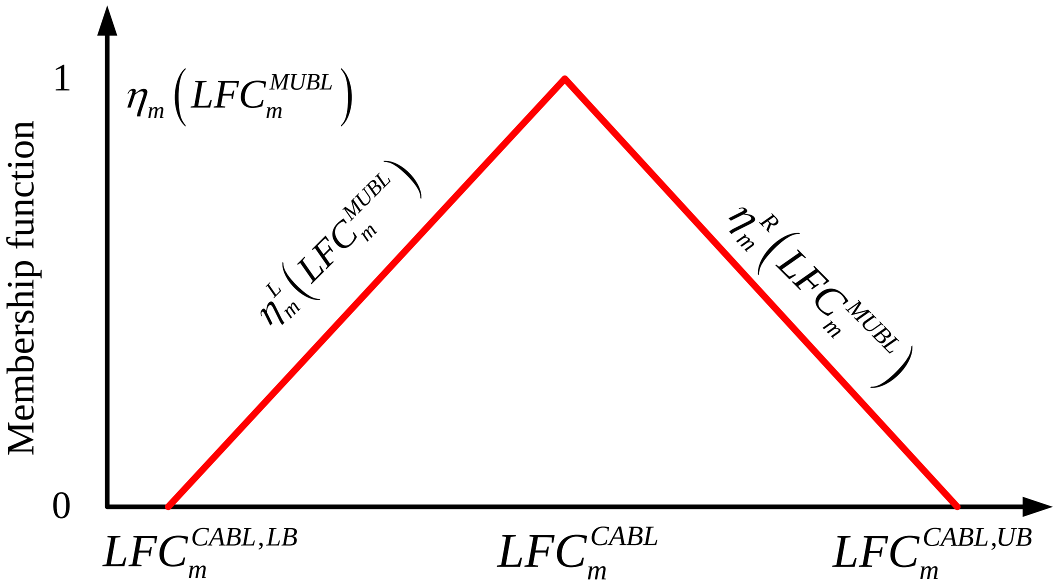

Figure S3. Definition of triangular membership function, $\eta_m\left(LFC_m^{MUBL}\right)$.

Solving Procedures for NHDE Algorithm

TLOP is a multi-objective mixed-integer optimization problem. The fuzzy equal objectives are used to evaluate the flux alternations. Each fuzzy equal objective can be quantified by eliciting a membership function. In this study, the membership function is a combination of the left-hand and right side line membership functions, as shown in Fig. S2. The mathematical expression is formulated as follows:

$$\eta_m\left(LFC_m^{MUBL}\right)=\begin{cases}0, & LFC_m^{MUBL}<LFC_m^{CABL,LB}\\ \dfrac{LFC_m^{MUBL}-LFC_m^{CABL,LB}}{LFC_m^{CABL}-LFC_m^{CABL,LB}}, & LFC_m^{CABL,LB}\le LFC_m^{MUBL}<LFC_m^{CABL}\\ 1, & LFC_m^{MUBL}=LFC_m^{CABL,LB}\\ \dfrac{LFC_m^{CABL,UB}-LFC_m^{MUBL}}{LFC_m^{CABL,UB}-LFC_m^{CABL}}, & LFC_m^{CABL}<LFC_m^{MUBL}\le LFC_m^{CABL,UB}\\ 0, & LFC_m^{MUBL}>LFC_m^{CABL,UB}\end{cases} \tag{S1}$$

where $LFC_m^{CABL,LB}$ and $LFC_m^{CABL,UB}$ are the lower and upper bounds of the logarithmic fold change (LFC_m) of the synthesis rates in cancer and basal states for m^{th} metabolite. The membership functions for all metabolites are summed up to form

the decision grade as $\eta_{MUBL} = \frac{1}{M}\sum_{m=1}^{M} \eta_m \left(LFC_m^{MUBL} \right)$. The multi-objective functions in Equation S1 can be combined to form a fitness in the NHDE algorithm to evaluate which the individuals survive in the next generation. The fitness, η, for each individual is evaluated as

$$\eta_{CABL} = \eta_{MUBL} + \min\left\{ \eta_{MUBL}, \left(SR^{T,BL} + SR^{T_{rxn},BL} \right) / 2 \right\}$$

$$\eta_{CAHT} = \eta_{MUHT} + \min\left\{ \eta_{MUHT}, \left(SR^{T,HT} + SR^{T_{rxn},HT} \right) / 2 \right\}$$

$$\eta = \min\left\{ \eta_{CABL}, \eta_{CAHT} \right\}$$

Algorithm S1. Basic operation of the NHDE algorithms

1. Representation and initialization

$$(\mathbf{z}^0)_i = \text{uniformInt}(\mathbf{z}^{\min}, \mathbf{z}^{\max}),\ i = 1, \dots, N_p$$

Each individual is generated by an integer random number between $\mathbf{z}_{\min}$ and $\mathbf{z}_{\max}$ with uniform distribution

2. Mutation with rounding operation

$$(\hat{\mathbf{z}}^G)_i = \text{INT}\left\{ (\mathbf{z}^G)_p + \rho^G \left[(\mathbf{z}^G)_j - (\mathbf{z}^G)_k + (\mathbf{z}^G)_l - (\mathbf{z}^G)_m \right] \right\}$$

3. Crossover operation

$$z_{ji}^G = \begin{cases} z_{ji}^{G-1}, & \text{if a random number} > C_R \\ \hat{z}_{ji}^G, & \text{otherwise},\ j = 1, \dots, n;\ i = 1, \dots, N_p \end{cases}$$

4. Restriction operation

$$z_{ji}^G = \begin{cases} z_{ji}^G, & z_{ji}^G \in [z_j^{\min}, z_j^{\max}] \\ \text{uniformInt}(z_j^{\min}, z_j^{\max}), & z_{ji}^G \notin [z_j^{\min}, z_j^{\max}] \end{cases}$$

5. Selection and evaluation
   (a) For each individual, solve FBA in the inner optimization problem by a LP solver

$$\begin{cases} \text{FBA problem} \\ \max_{\mathbf{v}_{f/b}} \left(w_{biomass} v_{biomass} + w_{ATP} v_{ATP} \right) \\ \text{subject to} \\ \quad \mathbf{N}^{BL} \left(\mathbf{v}_f - \mathbf{v}_b \right) = \mathbf{0} \\ \quad v_{f/b,i}^{LB,MU} \le v_{f/b,i} \le v_{f/b,i}^{UB,MU},\ z_i \in \Omega^{MU} \\ \quad v_{f/b,j}^{LB} \le v_{f/b,j} \le v_{f/b,j}^{UB},\ z_j \notin \Omega^{MU} \end{cases}$$

(b) Solve UFD in the inner optimization problem by a QCP solver

$$\begin{cases} \text{UFD problem} \\ \min_{\mathbf{v}_{f/b}} \sum_{i \in \Omega^{Int}} \left(v_{f,k}\right)^2 + \left(v_{b,k}\right)^2 \\ \text{subject to} \\ \quad \mathbf{N}^{BL}\left(\mathbf{v}_f - \mathbf{v}_b\right) = \mathbf{0} \\ \quad v_{f/b,i}^{LB,MU} \le v_{f/b,i} \le v_{f/b,i}^{UB,MU},\ z_i \in \Omega^{MU} \\ \quad v_{f/b,j}^{LB} \le v_{f/b,j} \le v_{f/b,j}^{UB},\ z_j \notin \Omega^{MU} \\ \quad \left(w_{ATP} v_{ATP} + w_{biomass} v_{biomass}\right) \ge \\ \quad \left(w_{ATP} v_{ATP}^{*} + w_{biomass} v_{biomass}^{*}\right) \end{cases}$$

(c) Compute the fitness for each feasible solution

$$fitness_i = \eta_i + \text{penalty}$$

A penalty is added to the fitness, if FBA/UFD is infeasible.

6. Migration operation performed naturally or enforced if necessary

$$(\mathbf{z}^G)_i = \text{uniformInt}(\mathbf{z}^{\min}, \mathbf{z}^{\max}),\ \text{if } \zeta \le \varepsilon = [0,1]$$

7. Repeat steps 2 to 6

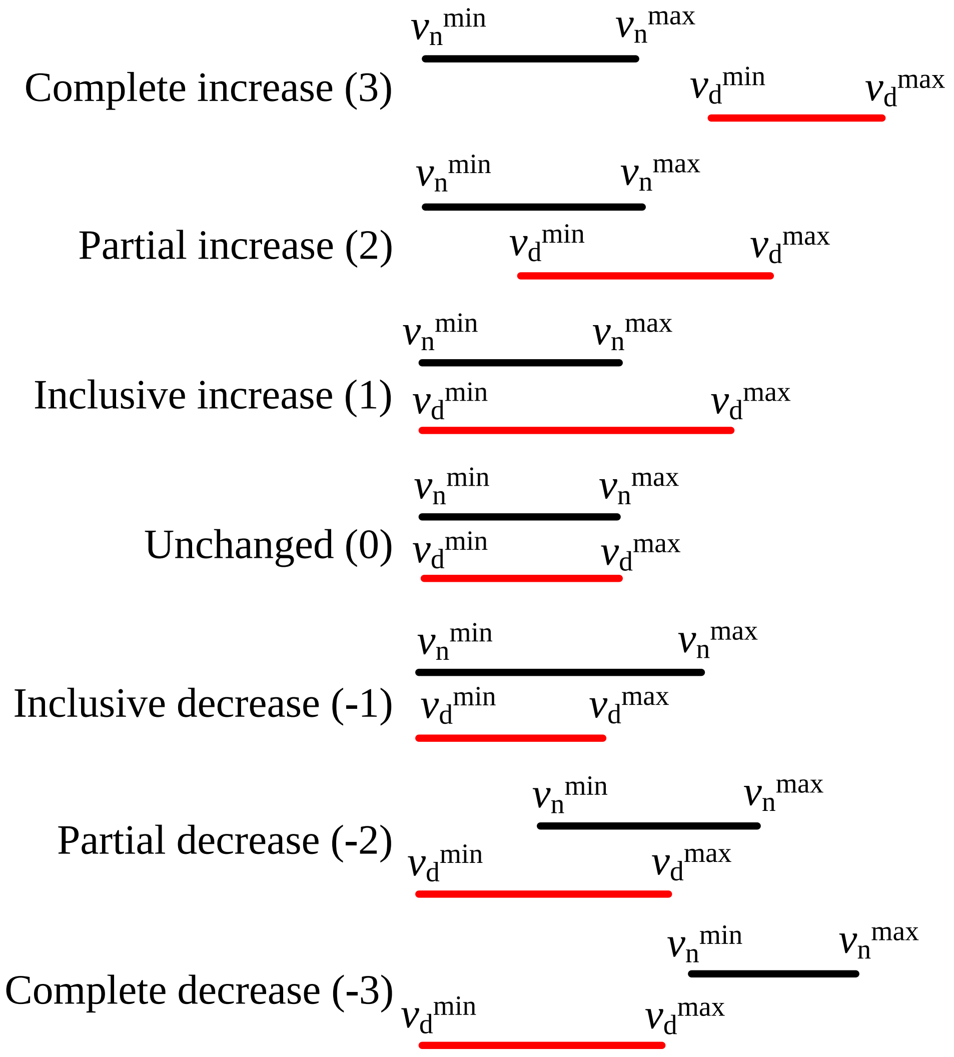

Figure S4: Categories of flux variance between the normal and mutant model. The black line indicates the flux interval of the normal model, and the red line indicates that of the mutant/cancer model. Number in the brackets is the indicator to denote each category.

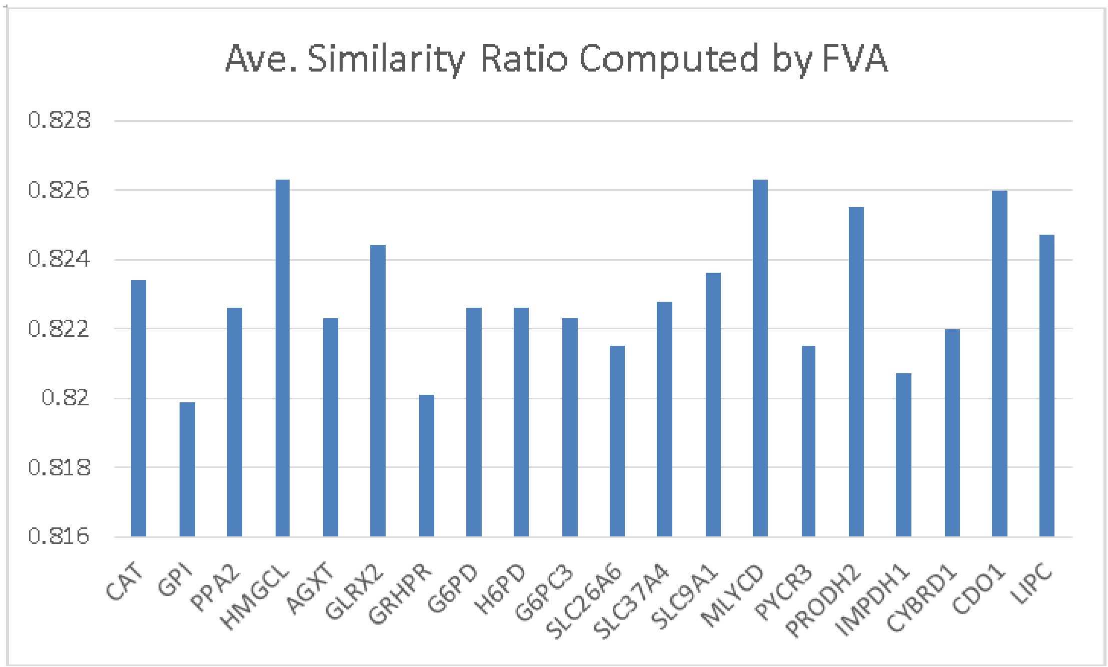

Figure S5: Average similarity ratios computed by FVA. Each mutant is compared with the template as shown in Figure 6.
